# Supplementary figures and images for: Successful introgression of wMel Wolbachia into Aedes aegypti populations in Fiji, Vanuatu and Kiribati
Source: PLoS Negl Trop Dis. 2024 Mar 14;18(3):e0012022. doi: 10.1371/journal.pntd.0012022 (PMC10980184; doi:10.1371/journal.pntd.0012022)

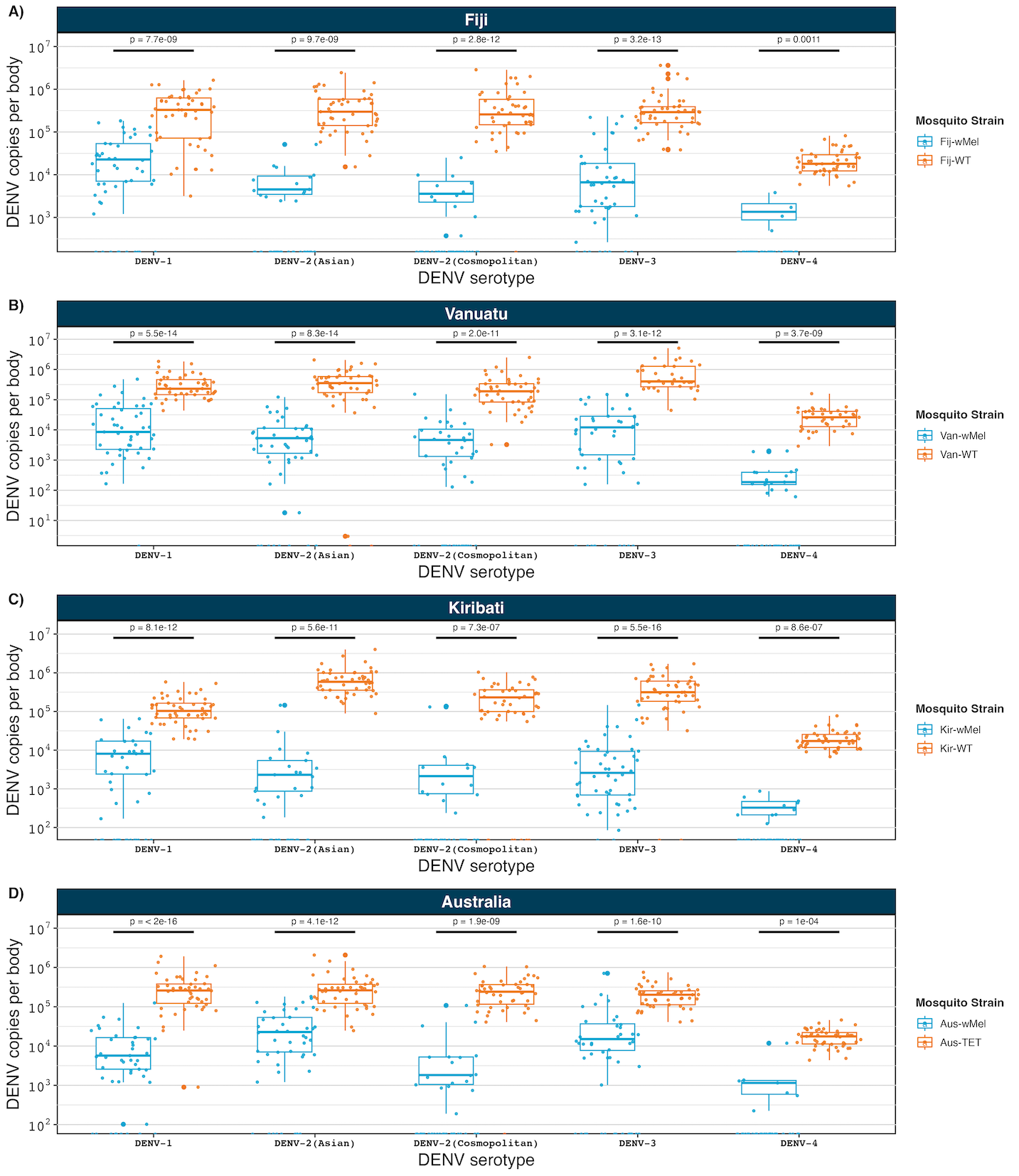

Supplement: S1 Fig — All mosquitoes were aged for 6–7 days prior to intrathoracic injection with DENV. Fifty mosquitoes were used for each data point but some died prior to testing (S3 Table). DENV copy number was determined 7 days post injection using qRT-PCR. All wMel Ae. aegypti lines had a significant reduction in DENV viral RNA concentration (Wilcoxon rank-sum est). A) Fiji release strain, Fij-wMel, and wild-derived control, Fij-WT,vector competence. B) Vanuatu release strain, Van-wMel, and wild-derived control, Van-WT, vector competence. C) Kiribati release strain, Kir-wMel, and wild-derived control, Kir-WT, vector competence. D) Australian Cairns strain, Aus-wMel, and tetracycline cured control, Aus-TET, vector competence. Data are shown as the median DENV copies per mosquito (thick line) ± interquartile ranges (box), extended by the whiskers indicating 1.5× the interquartile range, with dots indicating outliers. Individual data points are included as smaller partially opaque points. Data from uninfected mosquitoes are not included in the median estimates (S3 Table). (TIFF) [file pntd.0012022.s001.tiff]

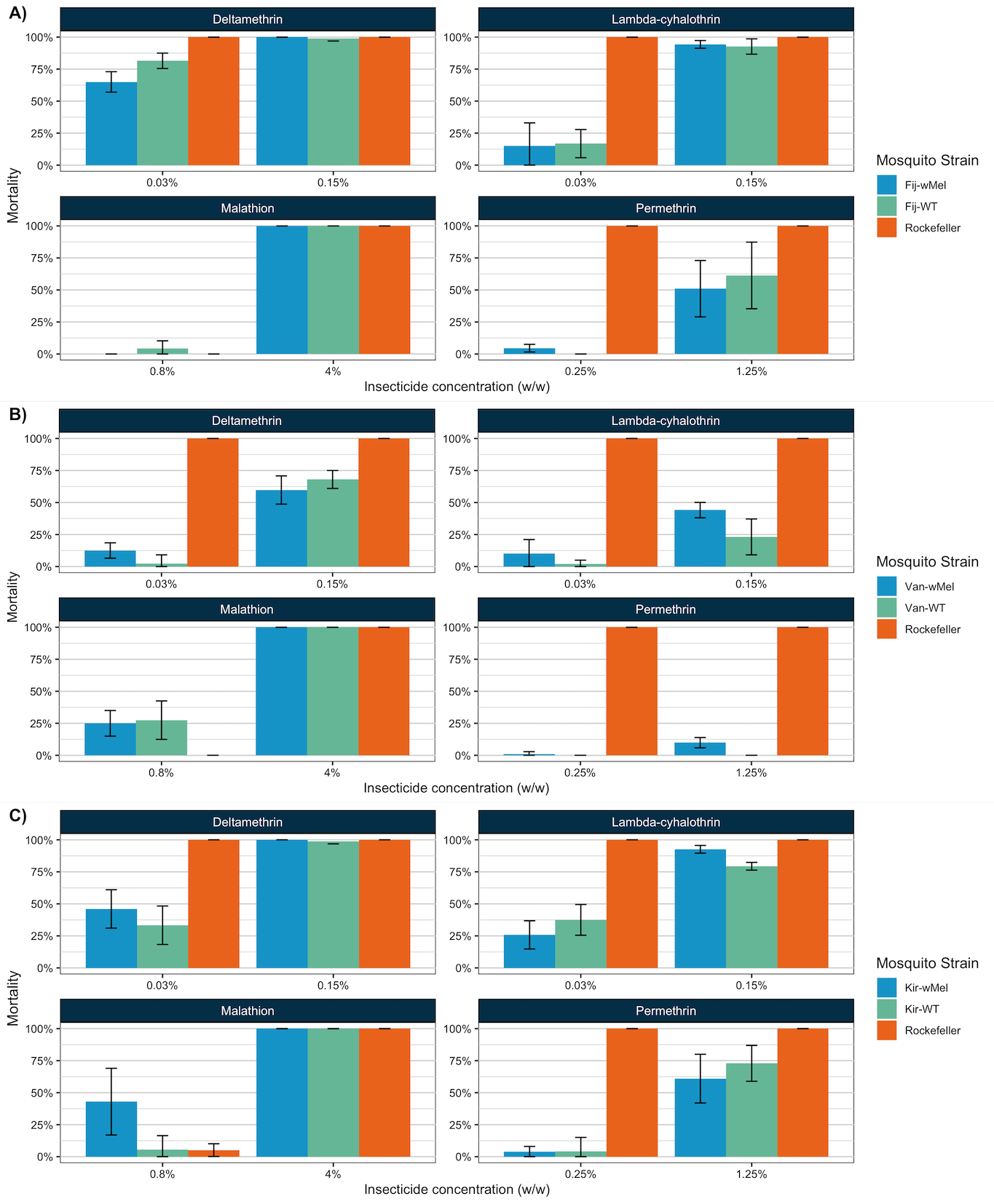

Supplement: S2 Fig — A) Fiji release strain IR profile. B) Vanuatu release strain IR profile. C) Kiribati release strain IR profile. Each data point is the mean of five biological replicates (± s.d.) using approximately 20 mosquitoes per replicate. (TIFF) [file pntd.0012022.s002.tiff]

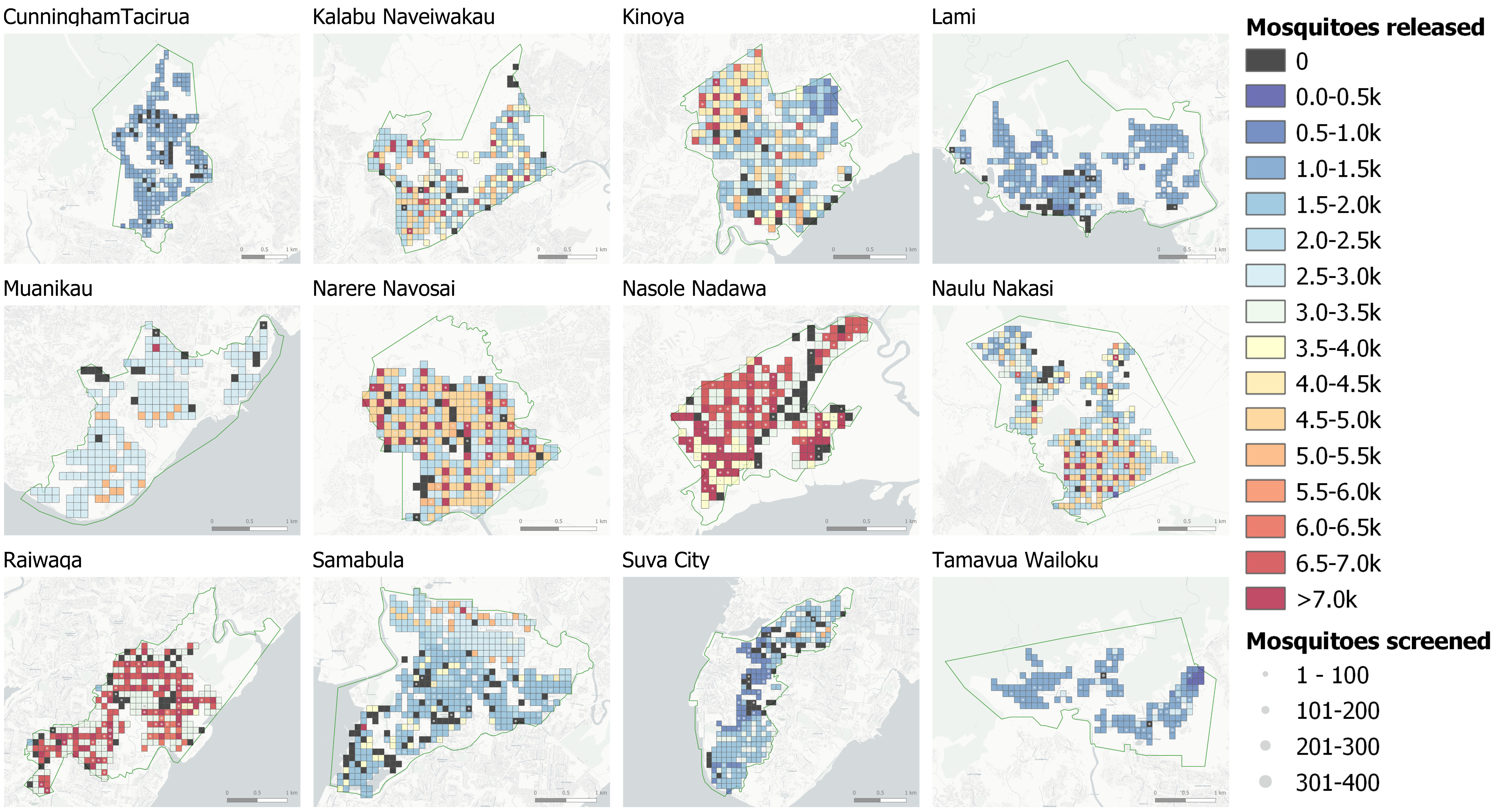

Supplement: S3 Fig — Each release area was divided into a grid with 100 x 100 meter squares. Grid squares lacking mosquito releases were omitted. Release gradient was determined by using GPS coordinates of each release event and assigning the number of wMel-infected mosquitos to a corresponding grid square. Monitoring numbers were determined in the same way. Map produced in QGIS version 3.16.1 using boundaries aggregated from the enumeration area boundaries freely available from the Pacific Data Hub (https://pacificdata.org/data/dataset/2007_fji_phc_admin_boundaries) and OpenMapTiles basemap layer (https://openmaptiles.org/) with CARTO light design (https://carto.com/)). (PNG) [file pntd.0012022.s003.png]

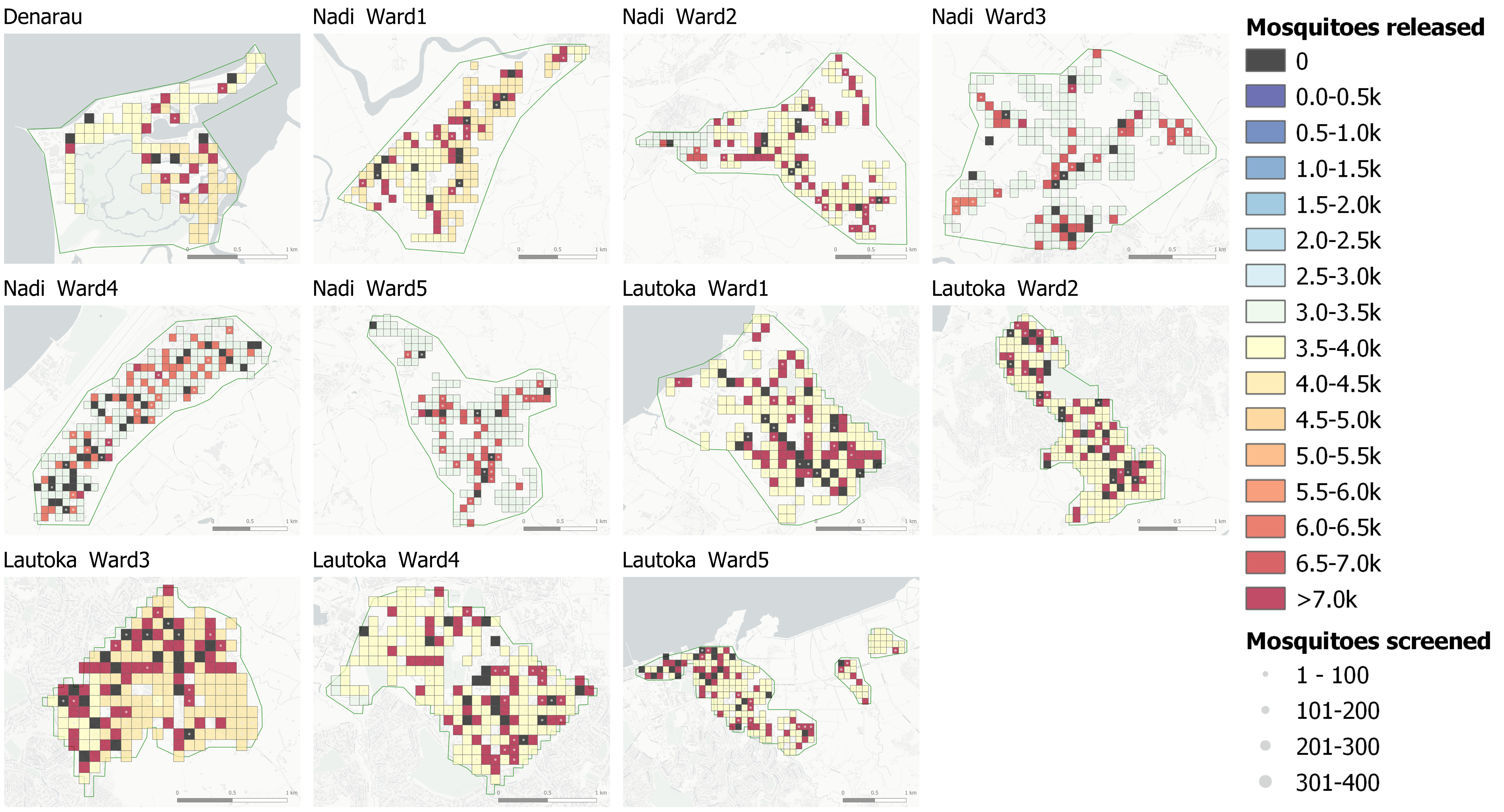

Supplement: S4 Fig — Each release area was divided into a grid with 100 x 100 meter squares. Grid squares lacking mosquito releases were omitted. Release gradient was determined by using GPS coordinates of each release event and assigning the number of wMel-infected mosquitos to a corresponding grid square. Monitoring numbers were determined in the same way. Map produced in QGIS version 3.16.1 using boundaries aggregated from the enumeration area boundaries freely available from the Pacific Data Hub (https://pacificdata.org/data/dataset/2007_fji_phc_admin_boundaries) and OpenMapTiles basemap layer (https://openmaptiles.org/) with CARTO light design (https://carto.com/)). (PNG) [file pntd.0012022.s004.png]

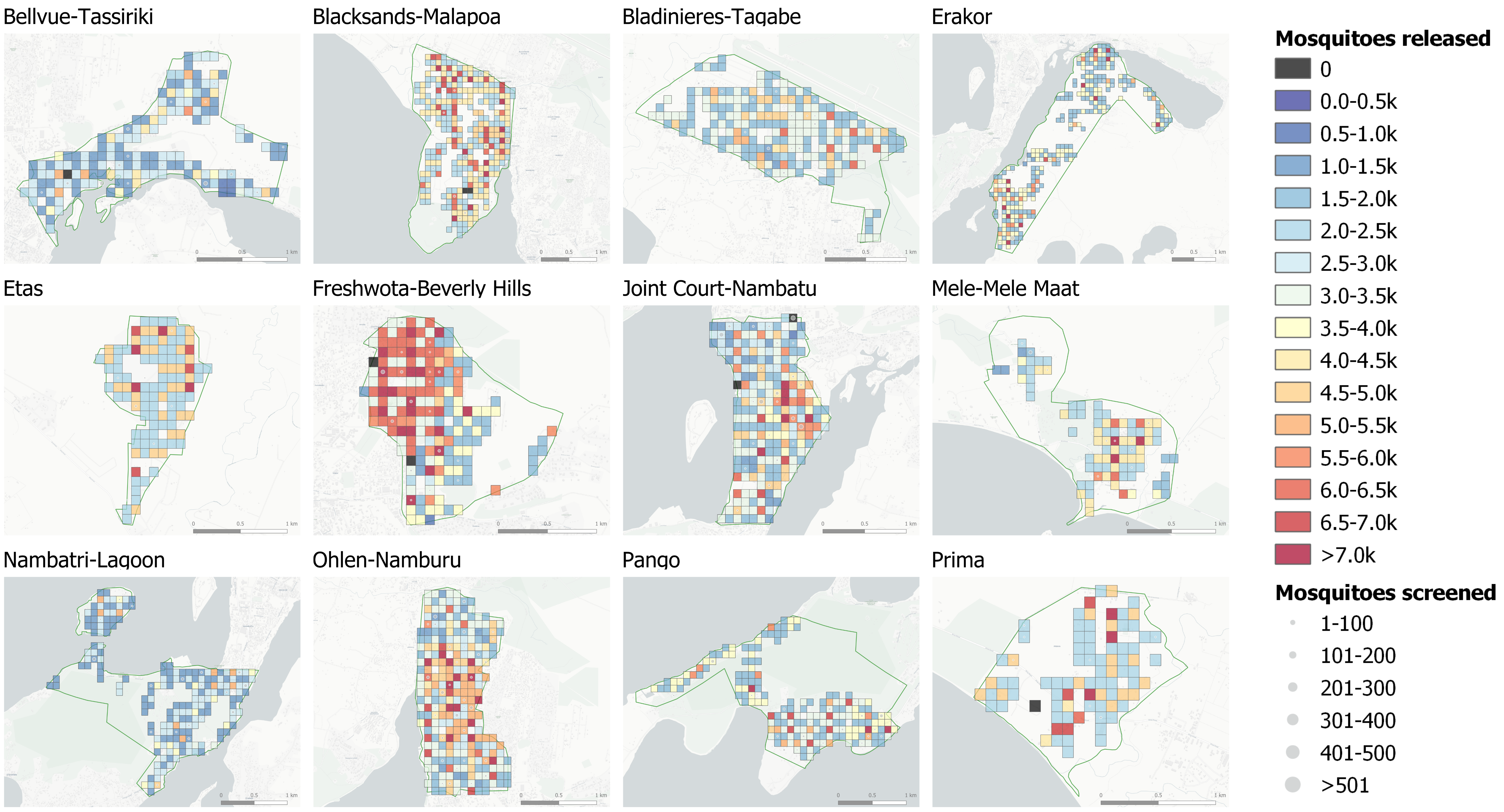

Supplement: S5 Fig — Each release area was divided into a grid with 100 x 100 meter squares. Grid squares lacking mosquito releases were omitted. Release gradient was determined by using GPS coordinates of each release event and assigning the number of wMel-infected mosquitos to a corresponding grid square. Monitoring numbers were determined in the same way. Map produced in QGIS version 3.16.1 using boundaries aggregated from the enumeration area boundaries freely available from the Pacific Data Hub (https://pacificdata.org/data/dataset/2016_vut_phc_admin_boundaries) and OpenMapTiles basemap layer (https://openmaptiles.org/) with CARTO light design (https://carto.com/)). (PNG) [file pntd.0012022.s005.png]

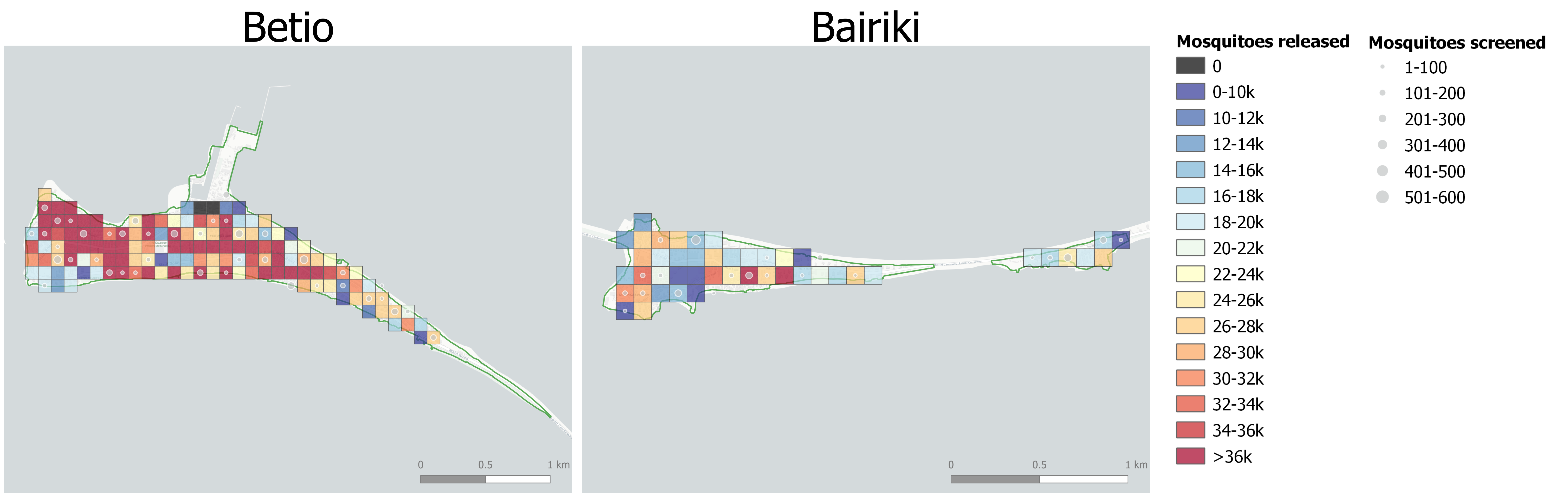

Supplement: S6 Fig — Each release area was divided into a grid with 100 x 100 meter squares. Grid squares lacking mosquito releases were omitted. Release gradient was determined by using GPS coordinates of each release event and assigning the number of wMel-infected mosquitos to a corresponding grid square. Monitoring numbers were determined in the same way. Map produced in QGIS version 3.16.1 using the enumeration area boundaries freely available from the Pacific Data Hub (https://pacificdata.org/data/dataset/2010_kir_phc_admin_boundaries) and OpenMapTiles basemap layer (https://openmaptiles.org/) with CARTO light design (https://carto.com/)). (PNG) [file pntd.0012022.s006.png]

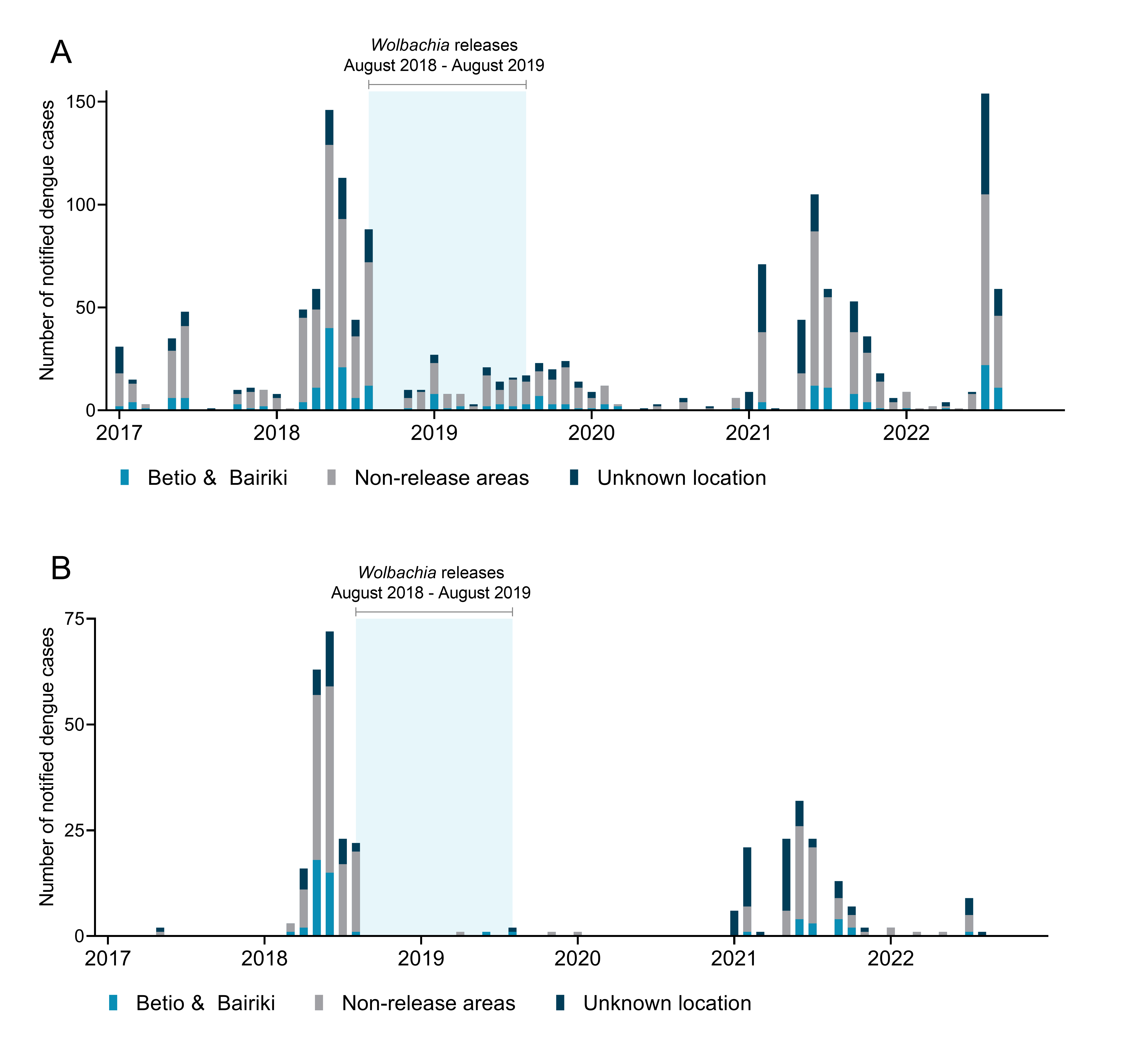

Supplement: S7 Fig — Cases with an ‘address’ location recorded were classified either as resident in the Wolbachia-release areas of Betio and Bairiki or in non-release areas (all locations other than Betio and Bairiki). Laboratory-confirmed dengue cases include those with a positive dengue NS1 and/or IgM diagnostic test result recorded. Blue shading indicates the Wolbachia release period. (TIF) [file pntd.0012022.s007.tif]
