# Supplementary material for: Successful introgression of wMel Wolbachia into Aedes aegypti populations in Fiji, Vanuatu and Kiribati
Source: PLoS Negl Trop Dis. 2024 Mar 14;18(3):e0012022. doi: 10.1371/journal.pntd.0012022 (PMC10980184; doi:10.1371/journal.pntd.0012022)
Supplement: S1 Table — (DOCX) [file pntd.0012022.s008.docx]

**S1 Table. Regulatory Permits for Pacific Releases.** Authority refers to the issuing authority for a given country. Australian permits are listed under general as their operation was not dependent on *Ae. aegypti* country of origin.

| **Purpose** | **Permit ID** | **Authority** |
| --- | --- | --- |
| **General** | | |
| Import | #PWS 2015-AU-001859 | Australian Government - Department of Environment |
| Import | 0000996845 | Australian Government - Department of Agriculture and Water Resources |
| Import | 0002334818 | Australian Government - Department of Environment |
| **Fiji** | | |
| Import | SUV-10076/18 | Biosecurity Authority Fiji |
| Import | SUV-02/18 | Biosecurity Authority Fiji |
| Import | SUV-10995/19 | Biosecurity Authority Fiji |
| Import | SUV-11304/19 | Biosecurity Authority Fiji |
| Import | SUV-11638/19 | Biosecurity Authority Fiji |
| Import | SUV-12014/19 | Biosecurity Authority Fiji |
| Premises Approval | BAP lm 320-05181 | Biosecurity Authority Fiji |
| **Vanuatu** | | |
| Import | LAIP#001/201 | Biosecurity Vanuatu |
| Export | VBDC/06030EN/cf | Government of the Republic of Vanuatu - Public Health Services |
| **Kiribati** | | |
| Import | 02/2018 | Kiribati Ministry of Environment, Lands & Agriculture Development Division of Agriculture |
| Transit | TS69/19 | Biosecurity Authority Fiji |
| Transit | TS95/19 | Biosecurity Authority Fiji |
| Transit | TS105/18 | Biosecurity Authority Fiji |
| Transit | TS145/18 | Biosecurity Authority Fiji |

### 
