# Supplementary material for: Successful introgression of wMel Wolbachia into Aedes aegypti populations in Fiji, Vanuatu and Kiribati
Source: PLoS Negl Trop Dis. 2024 Mar 14;18(3):e0012022. doi: 10.1371/journal.pntd.0012022 (PMC10980184; doi:10.1371/journal.pntd.0012022)
Supplement: S2 Table — (DOCX) [file pntd.0012022.s009.docx]

**S2 Table. Pre-release Mosquito Strain Health Checks.** Health checks were performed as previously described [[29]](https://www.zotero.org/google-docs/?ySjyYx). s.d. is standard deviation.

| **Release line** | **Characteristic** | **Description** |
| --- | --- | --- |
| Fiji-*w*Mel | Backcrossing source | Wild-type *Ae. aegypti* collected in Suva, Fiji |
|  | Backcrossing method | Males from the Suva wild-derived line were backcrossed with females from the Cairns *Ae. aegypti* *w*Mel-infected line for six generations, as previously described [26,27]. |
|  | Fecundity | Fiji-wMel: Eggs per Iso-female; *w*Mel-infected female x *w*Mel-infected male; 50 females; 81 ± 35(s.d.) |
|  |  | Fiji-WT: Eggs per Iso-female; wMel-infected female x wMel-infected male; 50 females; 76 ± 43 (s.d.) |
|  | Hatch rate | Fiji-wMel: Percentage of eggs that hatched per iso-female; *w*Mel-infected female x *w*Mel-infected male; 50 females. Result; 57.6 ± 37.1% (s.d.); 8 females with 0% hatch rate |
|  |  | Fiji-WT: Percentage of hatched eggs per iso-female; wMel-infected female x wMel-infected male; 50 females. Result; 68.89 ± 33.8 % (s.d.); 6 females with 0% hatch rate |
|  | Cytoplasmic Incompatibility | Percentage of hatched eggs per iso-female; 50 females; uninfected female x *w*Mel-infected male. Result; 0% – of eggs hatched from any of the 50 females |
|  | Maternal transmission | Percentage of hatched offspring per iso-female with *Wolbachia*; 38 *w*Mel-infected females mated with uninfected males; ~30 offspring (larvae) tested per female. Result; 100% *w*Mel prevalence in offspring |
|  | Fecundity under mass production conditions | N=41 batches of mass produced eggs; estimated mean eggs per female across all batches; 30.9 ± 12 (s.d.) |
| Van-*w*Mel | Backcrossing source | Wild-type *Ae. aegypti* collected in Port Vila, Vanuatu |
|  | Backcrossing method | Males from the Port Vila wild-derived line were backcrossed with females from the Cairns *Ae. aegypti* *w*Mel-infected line for six generations, as previously described [26,27]. |
|  | Fecundity | Van-*w*Mel: Eggs per Iso-female; *w*Mel-infected female x *w*Mel-infected male; 50 females; 38.92 ± 39.79 (s.d.) |
|  |  | Van-WT: Eggs per Iso-female; wMel-infected female x wMel-infected male; 46 females; 65.66 ± 34.83 (s.d.) |
|  | Hatch rate | Van-wMel: Percentage of hatched eggs per iso-female; *w*Mel-infected female x *w*Mel-infected male; 49 females; 33.76 ± 39.63 % (s.d.); 21 females with 0% hatch rate |
|  |  | Van-WT: Percentage of hatched eggs per iso-female; wMel-infected female x wMel-infected male; 46 females; 65.97 ± 37.47 % (s.d.); 6 females with 0% hatch rate |
|  | Cytoplasmic Incompatibility | Percentage of hatched eggs per iso-female; 50 females; uninfected female x *w*Mel-infected male;  5.67 ± 21%; only four females produced viable offspring; all but one offspring screened (n=217) were *Wolbachia* negative |
|  | Maternal transmission | Percentage of offspring from iso-females infected with Wolbachia; 39 wMel-infected females mated with uninfected males; ~30 offspring screened per female. Result; 97.5% ; 1 female with 0 Wolbachia infected offspring; total 931 larvae screened with 912 wMel positive |
|  | Fecundity under mass production conditions | N=37 batches of mass produced eggs; estimated mean eggs per female across all batches; 29 ± 13 (s.d.) |
| Kir-*w*Mel | Backcrossing source | Wild-type *Ae. aegypti* collected in South Tarawa, Kiribati |
|  | Backcrossing method | Males from the South Tarawa wild-derived line were backcrossed with females from the Cairns *Ae. aegypti* *w*Mel-infected line for six generations, as previously described [26,27]. |
|  | Fecundity | Kir-wMel: Eggs per iso-female; 48 females; *w*Mel-infected female x *w*Mel-infected male; 80.85 ± 29.65 (s.d.) |
|  |  | Kir-WT: Eggs per Iso-female; wMel-infected female x wMel-infected male; 50 females; 83.44 ± 31.2 (s.d.) |
|  | Hatch rate | Kir-wMel: Percentage of hatched eggs per iso-female; *w*Mel-infected female x *w*Mel-infected male; 48 females; 57.89 ± 33.21% (s.d.); 4 females with 0% hatch rate |
|  |  | Kir-WT: Percentage of hatched eggs per iso-female; wMel-infected female x wMel-infected male; 50 females; 84.71 ± 30.55 % (s.d.); 2 females with 0% hatch rate |
|  | Cytoplasmic Incompatibility | Percentage of hatched eggs per iso-female; 50 females; uninfected female x *w*Mel-infected male; 0.04 ± 0.29%; 1 females produced a single offspring; |
|  | Maternal transmission | Percentage of offspring from iso-females infected with *Wolbachia*; 48 *w*Mel-infected females mated with uninfected males; ~30 offspring screened per female. Result; 99.49; total 1302 larvae screened with 1295 *w*Mel positive |
|  | Fecundity under mass production conditions | N=48 batches of mass produced eggs; estimated mean eggs per female across all batches; 5.3 ± 2.8 (s.d.) |
