# Supplementary material for: Successful introgression of wMel Wolbachia into Aedes aegypti populations in Fiji, Vanuatu and Kiribati
Source: PLoS Negl Trop Dis. 2024 Mar 14;18(3):e0012022. doi: 10.1371/journal.pntd.0012022 (PMC10980184; doi:10.1371/journal.pntd.0012022)
Supplement: S3 Table — (DOCX) [file pntd.0012022.s010.docx]

**S3 Table. DENV Prevalence in *Wolbachia*-infected Mosquitoes.** All mosquitoes were aged for 6 – 7 days prior to intrathoracic injection with DENV. DENV copy number was determined 7 days post injection using qRT-PCR. Uninfected indicates DENV copies below the level of detection.

|  | **DENV-Infected Mosquitoes by Serotype –**  **Infected Mosquitoes (Total Mosquitoes Tested)** | | | | |
| --- | --- | --- | --- | --- | --- |
| **Mosquito Strain** | **DENV-1** | **DENV-2 (Asian)** | **DENV-2 (Cosmopolitan)** | **DENV-3** | **DENV-4** |
| **Fiji** | | | | | |
| Fij-WT | 48 (48) | 47 (47) | 45 (46) | 47 (47) | 47 (47) |
| Fij-*w*Mel | 38 (48) | 15 (48) | 12 (48) | 39 (48) | 4 (47) |
| **Vanuatu** | | | | | |
| Van-WT | 46 (46) | 46 (48) | 47 (47) | 32 (32) | 39 (39) |
| Van-*w*Mel | 47 (48) | 39 (48) | 28 (48) | 37 (47) | 17 (48) |
| **Kiribati** | | | | | |
| Kir-WT | 50 (50) | 48 (48) | 40 (47) | 45 (47) | 47 (47) |
| Kir-*w*Mel | 27 (45) | 21 (43) | 14 (46) | 46 (48) | 10 (47) |
| **Australia** | | | | | |
| Aus-TET | 47(47) | 47(47) | 46(47) | 47(47) | 47(47) |
| Aus-*w*Mel | 37(47) | 38(47) | 18(48) | 32(48) | 7(48) |
