# Supplementary material for: Successful introgression of wMel Wolbachia into Aedes aegypti populations in Fiji, Vanuatu and Kiribati
Source: PLoS Negl Trop Dis. 2024 Mar 14;18(3):e0012022. doi: 10.1371/journal.pntd.0012022 (PMC10980184; doi:10.1371/journal.pntd.0012022)
Supplement: S4 Table — (DOCX) [file pntd.0012022.s011.docx]

**S4 Table. *Wolbachia (w*Mel*)*-infected *Ae. aegypti* Mosquito Release Numbers.** Reporting area is the entire zone of each deployment. Release Area is space within a reporting area, excluding areas such as parks, airports and industrial zones, that received *w*Mel-infected mosquito deployments. Release weeks are the total number of weeks that release occurred in.

| **Location** | **Reporting area**  **(km^2^)** | **Release area**  **(km^2^)** | **Residents** | **First Release** | **Last Release** | **Release weeks** | **Total mosquitoes released** | **Release average per week** | **Total mosquitoes per km^2^** | **Total mosquitoes per inhabitant** |
| --- | --- | --- | --- | --- | --- | --- | --- | --- | --- | --- |
| **Fiji – Suva* (Total Reporting Area:72.62km^2^; Total Residents:242,817)** | | | | | | | | | | |
| Cunningham Tacirua | 7.1 | 2.97 | 24808 | Sep, 2018 | Nov, 2018 | 10 | 379,033 | 3,7903 | 127,620 | 15 |
| Kalabu Naveiwakau | 5.08 | 2.84 | 22369 | Sep, 2018 | Jan, 2019 | 19 | 947,615 | 4,9874 | 333,667 | 42 |
| Kinoya | 4.3 | 2.64 | 21415 | Sep, 2018 | Jan, 2019 | 16 | 903,667 | 5,6479 | 342,298 | 42 |
| Lami | 9.39 | 2.8 | 16989 | Jul, 2018 | Oct, 2018 | 12 | 340,045 | 2,8337 | 121,445 | 20 |
| Muanikau | 4.46 | 1.89 | 6170 | Oct, 2018 | Apr, 2019 | 17 | 574,792 | 3,3811 | 304,123 | 93 |
| Narere Navosai | 4 | 2.97 | 28534 | Oct, 2018 | Apr, 2019 | 18 | 1,258,347 | 69,908 | 423,686 | 44 |
| Nasole Nadawa | 3.2 | 2.69 | 21323 | Oct, 2018 | Apr, 2019 | 25 | 1,309,772 | 5,2391 | 486,904 | 61 |
| Naulu Nakasi | 8.3 | 3.83 | 26557 | Oct, 2018 | Apr, 2019 | 16 | 1,276,129 | 79,758 | 333,193 | 48 |
| Raiwaqa | 5.9 | 2.52 | 26743 | Oct, 2018 | Apr, 2019 | 24 | 1,712,725 | 71,364 | 679,653 | 64 |
| Samabula | 8.2 | 4.36 | 27545 | Sep, 2018 | Apr, 2019 | 20 | 1,032,712 | 51,636 | 236,861 | 37 |
| Suva City | 5.3 | 3.45 | 14434 | Dec, 2018 | Apr, 2019 | 13 | 410,016 | 31,540 | 118,845 | 28 |
| Tamavua Wailoku | 7.39 | 1.58 | 5930 | Jul, 2018 | Oct, 2018 | 12 | 231,405 | 19,284 | 146,459 | 39 |
| **Fiji – Nadi (Total Reporting Area:27.17km^2^; Total Residents:42,833)** | | | | | | | | | | |
| Denarau | 3.19 | 1 | 294 | May, 2019 | Nov, 2019 | 23 | 397,065 | 17,264 | 397,065 | 1351 |
| Nadi Ward 1 | 4.3 | 2.05 | 5453 | May, 2019 | Nov, 2019 | 22 | 837,024 | 38,047 | 408,304 | 153 |
| Nadi Ward 2 | 5.4 | 2.56 | 10504 | May, 2019 | Nov, 2019 | 23 | 920,454 | 40,020 | 359,552 | 88 |
| Nadi Ward 3 | 5.8 | 2.22 | 10093 | May, 2019 | Nov, 2019 | 24 | 762,816 | 31,784 | 343,611 | 76 |
| Nadi Ward 4 | 4.1 | 2.56 | 7368 | May, 2019 | Nov, 2019 | 23 | 788,101 | 34,265 | 307,852 | 107 |
| Nadi Ward 5 | 4.38 | 2.28 | 9121 | May, 2019 | Nov, 2019 | 24 | 776,250 | 32,344 | 340,461 | 85 |
| **Fiji – Lautoka (Total Reporting Area:14.75km^2^; Total Residents:53,830)** | | | | | | | | | | |
| Lautoka Ward 1 | 3.6 | 2.13 | 10719 | May, 2019 | Nov, 2019 | 23 | 795,129 | 34,571 | 373,300 | 74 |
| Lautoka Ward 2 | 2.8 | 2.4 | 16233 | May, 2019 | Nov, 2019 | 25 | 934,560 | 37,382 | 389,400 | 58 |
| Lautoka Ward 3 | 2.4 | 2.24 | 10453 | May, 2019 | Nov, 2019 | 26 | 895,512 | 34,443 | 399,782 | 86 |
| Lautoka Ward 4 | 3.16 | 2.51 | 7290 | May, 2019 | Nov, 2019 | 26 | 961,270 | 36,972 | 382,976 | 132 |
| Lautoka Ward 5 | 2.79 | 2.28 | 9135 | May, 2019 | Nov, 2019 | 26 | 887,690 | 34,142 | 389,338 | 97 |
| **Vanuatu (Total Reporting Area:38.66km^2^; Total Residents:61,297)** | | | | | | | | | | |
| Bellvue-Tassiriki | 2.05 | 1.8 | 4424 | Oct, 2018 | Feb, 2019 | 13 | 39,4946 | 30,380 | 219,414 | 89 |
| Blacksands-  Malapoa | 5.15 | 3.13 | 11131 | Sep, 2018 | Feb, 2019 | 23 | 1,190,959 | 51,781 | 380,498 | 107 |
| Bladinieres-Tagabe | 3.29 | 2.23 | 6225 | Sep, 2018 | Jan, 2019 | 18 | 629,773 | 34,987 | 282,409 | 101 |
| Erakor | 6.53 | 2.86 | 3551 | Nov, 2018 | Mar, 2019 | 14 | 933,133 | 66,652 | 326,270 | 263 |
| Etas | 1.39 | 0.92 | 1126 | Dec, 2018 | Mar, 2019 | 14 | 378,348 | 27,025 | 411,248 | 336 |
| Freshwota-  Beverly Hills | 2.64 | 1.86 | 8685 | Aug, 2018 | Feb, 2019 | 19 | 817,210 | 43,011 | 439,360 | 94 |
| Joint Court-Nambatu | 2.5 | 2.47 | 6593 | Jul, 2018 | Dec, 2018 | 25 | 743,123 | 29,725 | 300,860 | 112 |
| Mele-Mele Maat | 2.56 | 0.82 | 805 | Nov, 2018 | Mar, 2019 | 13 | 278,024 | 21,386 | 339,054 | 345 |
| Nambatri-Lagoon | 3.59 | 1.93 | 3236 | Sep, 2018 | Feb, 2019 | 16 | 446,896 | 27,931 | 231,552 | 138 |
| Ohlen-Namburu | 2.78 | 2.4 | 12104 | Sep, 2018 | Jan, 2019 | 17 | 988,411 | 58,142 | 411,838 | 82 |
| Pango | 4.37 | 1.85 | 2693 | Oct, 2018 | Mar, 2019 | 15 | 589,209 | 39,281 | 318,491 | 219 |
| Prima | 1.81 | 0.76 | 724 | Oct, 2018 | Mar, 2019 | 15 | 274,770 | 18,318 | 361,539 | 380 |
| **Kiribati (Total Reporting Area:1.5km^2^; Total Residents Covered:30,104)** | | | | | | | | | | |
| Bairiki | 0.6 | 0.37 | 6006 | Oct, 2018 | Aug, 2019 | 29 | 1,063,709 | 36,680 | 2,874,889 | 177 |
| Betio | 1.5 | 1.05 | 24098 | Aug, 2018 | May, 2018 | 42 | 4,361,649 | 103,849 | 4,153,951 | 181 |

#### 
