# Supplementary material for: Successful introgression of wMel Wolbachia into Aedes aegypti populations in Fiji, Vanuatu and Kiribati
Source: PLoS Negl Trop Dis. 2024 Mar 14;18(3):e0012022. doi: 10.1371/journal.pntd.0012022 (PMC10980184; doi:10.1371/journal.pntd.0012022)
Supplement: Attachment — Submitted filename: simmons_reviewer_response (1).docx [file pntd.0012022.s012.docx]

*Reviewer #1: In relation to field monitoring survey, I reckon it is important to provide estimates of the Ae. aegypti population size prior and during the releases in order to assess the population density for efficient release design (as stated by the authors in the Discussion), but also for assessing the impact of the releases at level of local mosquito population size. This also represents an important aspect which should be assessed in the post-release survey of local communities (for example, the perceived estimate of mosquitoes circulating in release areas). In addition, data on the composition of the Aedes population (i.e. other sympatric species, like Ae. albopictus, Ae. polynesiensis) would also be very informative and useful to interpret the epidemiological impact of the releases. This monitoring can be done by placing ovitraps on release sites and by performing a comparative analysis with data from control sites. Have the authors collected this data?*

Author’s response; Unfortunately we did not perform detailed entomological surveys before or after *Wolbachia* deployment. It’s important for the reviewers to understand the context of the activities; these 3xPacific Island projects were funded through the Australian Government’s international aid program in response to the impact of regional and global Zika virus outbreaks (which led to the WHO declaration of a PHEIC in early 2016). As such, these projects were not “field experiments” but pragmatic deployments of an evidence-based intervention into at-risk, low-middle income communities. In summary, there was simply insufficient time in the schedule or funding in the budget to enable the detailed entomological surveys that we, and the reviewer, would have preferred.

*Could you comment on the choice of not including matching control (i.e. where no release of wMel-carrying Ae. aegypti occur) sites, potentially surrounding release sites. Is there a rational for this? Comparative epidemiological data and mosquito population density data from these sites would have been very important in this study.*

Author’s response; The goal of these deployments was pragmatic delivery of an evidence-based intervention. The scope of deployments was at the recommendation of respective Ministries of Health in each country and was deliberately aimed at maximising the beneficiary population. This meant “leaving out” a geographic area to serve as a control (counterfactual to the intervention area) was not considered an option. In Port Vila this meant deployment in the entire urban and peri-urban area; there is no other similar area on the island of Efate that could serve as a control. In Fiji, we deployed *Wolbachia* to the three largest population centres in the country; Suva, Nadi and Lautoka. There are no other urban area on the island of Viti Levu that could conceivably serve as a control area. In Kiribati ( Tarawa) there was no conceivable control area because of the geography of Kiribati (a collection of 33 islands/atolls in tens of thousands of square kilometres of ocean) and because more than half the population lives in Tarawa.

*Reviewer #2: Although the authors give a clear outline of the release sites in the three Islands of choice it is unclear to me why they did not included a detailed outline of control sites or was it the case that none were included? The benefit of geographically distinct control sites, where no mosquitoes are released and area is similar to release sites i.e. population structure of vector and host, geographical location, terrain, urban/rural etc, is that a more direct evaluation of the impact of wMel releases can be obtained. For example, direct comparison of dengue cases in release vs control sites could be made (although I appreciate this may not always be possible as stated by the authors there is often incomplete residential data for reported infections).*

Author’s response; As above, the goal of these deployments was pragmatic delivery of an evidence-based intervention. The scope of deployments was at the recommendation of respective Ministries of Health in each country and was deliberately aimed at maximising the beneficiary population. This meant “leaving out” a geographic area to serve as a control (counterfactual to the intervention area) was not considered an option. In Port Vila this meant deployment in the entire urban and peri-urban area; there is no other similar area on the island of Efate that could serve as a control. In Fiji, we deployed *Wolbachia* to the three largest population centres in the country; Suva, Nadi and Lautoka. There are no other urban area on the island of Viti Levu that could conceivably serve as a control area. In Kiribati (Tarawa) there was no conceivable control area because of the geography of Kiribati (a collection of 33 islands/atolls in tens of thousands of square kilometres of ocean) and because more than half the population lives in Tarawa.

*Can the authors comment on why when carrying out fitness cost there was no data obtained from corresponding parental lines? This at the very least would determine if the introgression of wMel into the local background or the genetic background of the local mosquitoes in general have fitness issues. For example, the apparent low fecundity rate of Van-wMel (38.92 ± 39.79 (s.d.)), if this true for the parental Van line?*

Author’s response; As requested, these wild-type controls have been added to Table S2. The Vanuatu-*w*Mel line had lower fertility and fecundity rates in these “fitness check” laboratory experiments than their wild-type counterparts. Whether these *w*Mel infected mosquitoes have lower fecundity or fertility in the field is unknown- if it did, it was obviously not a barrier to eventual *w*Mel introgression and fixation in the population in Vanuatu (or Fiji or Kiribati).

*Reviewer #3:*

*1. What is minimum recommendation for mosquito sample size post-release monitoring?*

*2. What is minumum trapping design to allow robust monitoring?*

*3. Was post monitoring done in any particular season - would that matter?*

*4. Is thoracic challenge sufficient for these kinds of studies?*

*5. Any idea of approximate release ratio Wolbach : wildtype? Why no pre-intervention baseline? This would be standard practice for any biological release and control program.*

Author’s response;

1. We try to sample a minimum of 100 *Ae. aegypti* per reporting area. The rationale for 100 is that if the measured value of the wMel prevalence is 50% then the binomial 95% confidence will be +/- 10%, a level of uncertainty that we consider acceptable. In the real world it’s not always possible to reach the target sample size and in each reporting area e.g. because of seasonal (low) abundance or workforce capacity.
2. There isn’t a minimum trapping design per se. Monitoring approaches need to be pragmatic. We generally aim for ~10-12 monitoring points per reporting area during the release phase and ~4 during the long term monitoring
3. Post release monitoring was timed for the rainy season when *Ae. aegypti* abundance would be highest and allow for easier field collection. There no reason to believe that seasonality in Pacific island countries will impact wMel prevalence.
4. Intrathoracic challenge, when it is benchmarked against a “gold standard” mosquito line that has been characterised epidemiologically (Carns wMel) is a pragmatic way of testing the
5. There was no pre-intervention baseline because this was not a research project. *Ae. aegypti* was understood to be present in all three locations because of published surveys and the respective Ministries of Health own surveillance of positive containers. We were not funded to perform a baseline survey because this was intended as a pragmatic public health intervention - and our Ministries of Health partners agreed with this approach.

--------------------

***Reviewer #1****: Datasets from the post-release monitoring are limited in some sites (such as Suva, Vanuatu), compared to others – is this related to a very low population density in those areas or to the applied collection methods? Why the authors have opted for adult collections (with traps or aspirators) over using ovitraps (this would have probably increased the number of individuals to be tested)?*

Author’s response; We have added a sentence to the Methods section to explain that the sampling density during the post-release monitoring was less than in the release periods and hence a lower yield of Ae. aegypti were caught. In all three countries BG traps were used for monitoring during the release phase. Long term monitoring was led by the respective Ministries of Health. For human resource reasons, the BG trap, or prokopak collections, were brief in duration (2-4 weeks) and with a lower density of BG traps (typically ~2-3 per km2 ), and hence the total yield of mosquitoes per month was smaller than in the active release periods, when the density of BG traps in Suva and Port Vila was ~3–10 per km^2^ and in South Tarawa was ~13–20 per km^2^. Suva, in general, had a lower *Ae. aegypti* abundance than Pt Vila and Tarawa. The use of BG traps over ovitraps in long term monitoring was to be consistent with the trapping methodology used during releases.

*I suggest to also include the phenotypic data of the original wMel line (Cairns Ae. aegypti wMel-infected line) and the wild-type line sharing the same genetic background, used for introgressions, where appropriate depending on the assay. This is important for comparing possible costs related to the genetic background. In relation to this point, reduced fecundity and hatching rate were observed in the Van-wMel line, compared to the other genetic backgrounds (Fecundity: 38.92 ± 39.79 (s.d.) Hatching: 49 females;33.76 ± 39.63 (s.d.); 21 females with 0% hatch rate). Does the Van-WT line have a similarly reduced fecundity and fertility?*

Author’s response; As mentioned above, fertility and fecundity data from the corresponding WT lines has been added to Table S2.

*In the Discussion, the authors associate the failure of introgression in some areas and the reduction of the geographic scope of the project with the low fecundity of the Kiribati line (“This was linked to generally lower fecundity of the Kiribati release line versus Fiji and Vanuatu lines”). However, looking at S2 Table. Pre-release Mosquito*

*Strain Health Checks, this line showed a relatively higher fecundity (80.85 ± 29.65 (s.d.) and hatch rate(57.89 ± 33.21% (s.d.)). Can the authors discuss this point?*

Author’s response; We have edited the text in the Discussion to clarify our message here. What we observed under mass production conditions was that the Kiribati line was the least fecund of the three lines. We don’t know why this was the case. Mass production conditions are different to the conditions under which initial fitness tests are performed; mass production involves higher density of mosquitoes per cage, multiple gonotropic cycles of egg production and occasional use of blood blends (human/horse) for blood feeding. The low fecundity of the Kiribati line under mass production conditions, coupled with space constraints in the insectary, thus limited our output of *w*Mel eggs to supply the Kiribati project. Thus, reducing geographic scope and focusing the available Kiribati mosquito release material into a smaller geographic area was the only practical option available to us. We have added details of the fecundity of each line under mass production conditions as a new row for each country line in Table S2

***Reviewer #2****: Can the authors comment on the low number of mosquitoes used to assess the long-term level of wMel within the populations?*

Author’s response; As per the answer to Reviewer #1 on this same question, we have added clarity to the Methods section to explain why fewer mosquitoes were caught in long term monitoring windows.

*The authors do not present data on Wolbachia density within each line and in comparison to the parental wMel line. Can the authors comment on if there are differences in Wolbachia density between the different genetic backgrounds?*

Author’s response; We didn’t test *w*Mel density because the vector competence results demonstrated highly significant restriction on DENV replication in the mosquito body that was equivalent to that obtained with the *w*Mel parental line used for the backcrossing (Cairns *w*Mel). Note we have now included the data for this Cairns *w*Mel line in Fig. S1 and Table S3.

*It would be beneficial for the authors to discuss why DENV challenges were carried out by intrathoracic injection and not via blood meal. Given the reduction in follow up data due to the Sars-CoV-2 pandemic with regards to dengue cases, clearly showing that each introgressed background line reduces dissemination of DENV would have made a stronger paper. To this end the authors should discuss why field mosquitoes were not collected from release sites and further challenged with DENV to show that the lines retained the ability to block the virus, this would have greatly improved the dataset particularly in light of the interruption to data analysis caused by the pandemic.*

Author’s response; Oral challenge experiments are difficult to perform reproducibly and require more resources (e.g. more mosquitoes, fresh blood, more staff time) than intrathoracic injections. We suggest that intrathoracic challenge is a reproducible approach to measuring the restriction on DENV replication in the mosquito body. We consider the parental wMel Cairns line as the “gold standard” and expect that newly created lines will have similar phenotypes in response to intrathoracic injections with DENV-1-4. Indeed this is what we observed with the 3x Pacific lines described here- Fig. S1 and Table S3. We have added additional justification for intrathoracic injections to the Methods section.

With respect to capturing back mosquitoes from the field and measuring their vector competence back in the laboratory - this was not feasible with the funding envelope we had available.

*In figure S1 The authors state that 50 mosquitoes were used for each datapoint. However the data points are unclear in many of the box plots. Can the authors comment on this? How many data points were used per group?*

Author’s response;

As suggested, the Figure S1 has been revised and now shows each of the datapoints in each experimental group.

***Reviewer #3****:*

*1. Fig 1-5. Address why it is not possible to give any estimate of variance around % wolbachia infected.*

Author’s response; We could show the variance around each point prevalence estimate of the *w*Mel infection level but it results in unreadable graphs because the graphs are already depicting *w*Mel prevalence over time as well as the number of *Ae. aegypti* caught per monitoring window.

*2. Why not include Fiji dengue data? Limitations re: spatial and temporal accuracy can't be that different to the other countries?*

Author’s response; The data from Fiji was not available for publication purposes because the Fiji Ministry of Health and Medical Services required a human ethics committee approval for the data to be published. Due to significant staff turnover in the Ministry of Health and Medical Services, we have not been able to work with a local principal investigator to secure this ethics approval. We hope to do so in the future.

--------------------

*Reviewer #1: The aim of the paper was to describe the introgression of Wolbachia wMel into the local populations of Ae. aegypti. The characterization of the basic life hostly parameters and viral blockage capacity of the newly introgressed lines were assessed. Although with some limitations, data on the field monitoring of Wolbachia prevalence in recapture mosquitoes were reported. Therefore, the overall conclusions of the study are supported by the data presented. Epidemiological data are not solid enough for drawing robust conclusions of the impact of the releases, however the public health outcome was not intended as the main focus of the study.*

*Nevertheless, I suggest to address the following points in the discussion section:*

*Phenotypic stability and viral blockage of some variants of wMel is also dependent on environmental conditions, such as temperature. Therefore, it is important to include details about the climate of the release areas and to evaluate whether such susceptibility could be a potential factor contributing to the reduced establishment and invasion ability of wMel-carrying mosquitoes in some areas.*

Author’s response; We agree climate can influence the probability of *w*Mel establishment and have added new text to the Discussion to highlight this. However, given that *w*Mel has established in nearly 100% of the targeted release areas in each country (including Kiribati despite a reduced geographic scope), we don’t think climate is a particularly relevant variable here. In the very small geographic areas where *w*Mel clearly did not establish, such as Denarau (Fiji) and Mele-Mele Maat (Vanuatu), there is no reason to think that the climate is different from the neighbouring, much larger, geographic areas, just a few kilometres away, where *w*Mel has established. In practical terms, what matters most are the temperatures in breeding sites, with high water temperatures known to have detrimental impacts on traits that promote *w*Mel introgression, such as *w*Mel maternal transmission and cytoplasmic incompatibility. This is obviously impossible to measure at scale and given the diversity of breeding sites, known and cryptic.

*Although being aware that viral infections via intra- thoracic injections and viral quantification using qPCRs on whole bodies are operationally easier for performing viral challenges using four different dengue serotypes, they represent a proxy with substantial limitations and biases for assessing the true ability of the mosquito lines to transmit arboviruses. Therefore, I recommend that these limitations of the study should be at least aknowledged and addressed in the text.*

Author’s response; Agreed. As suggested we have added text describing the operational advantages and limitations of using intrathoracic injections (versus oral challenge) in the Discussion section.

*Reviewer #2: When discussing potential reasons for failure of introgression the authors fail to discuss that wMel density and maternal transmission can be affected by environmental factors such as high temperatures. Although not necessarily the case here, it would be prudent to at least discuss it.*

Author’s response; As per the response above, we agree climate can influence the probability of *w*Mel establishment. However, given that *w*Mel has established in nearly 100% of the targeted release areas in each country (including Kiribati despite a reduced geographic scope), we don’t think climate is a relevant variable here. In the very small geographic areas where *w*Mel clearly did not establish, such as Denarau (Fiji) and Mele-Mele Maat (Vanuatu), there is no reason to think that the climate is different from the neighbouring, much larger, geographic areas, just a few kilometres away, where *w*Mel has established. In practical terms, what matters most are the temperatures in breeding sites, with high water temperatures known to have detrimental impacts on traits that promote *w*Mel introgression, such as *w*Mel maternal transmission and cytoplasmic incompatibility. This is obviously impossible to measure at scale and given the diversity of breeding sites, known and cryptic.

*The authors state that they reduced the number of release sites in Kiribati due to several reasons one being the reduced fecundity of the introgressed background line used in this area. However, the fecundity data presented does not appear to align with this: 80.85 ± 29.65 (s.d.)) for the Kir-wMel compared to say 38.92 ± 39.79 (s.d.) for the Van-wMel line. This would suggest that in fact the fecundity for Kir-wMel was in fact much better than for Van-wMel. Further to this the standard deviation for the Van-wMel line is extremely high. Can the authors comment on this and discuss if there are highly levels of variation and an abnormal distribution of data? If so, it may be more advisable to use the median rather than the mean and to state the range rather than the standard deviation.*

Author’s response;We have changed the text in the Discussion to clarify our message here. What we observed under mass production conditions was that the Kiribati line was the least fecund of the three lines. We don’t know why this was the case. Mass production conditions are different to the conditions under which initial fitness tests are performed; mass production involves higher density of mosquitoes per cage, multiple gonotropic cycles of egg production and occasional use of blood blends (human/horse) for blood feeding. The low fecundity of the Kiribati line under mass production conditions, coupled with space constraints in the insectary, thus limited our output of *w*Mel eggs to supply the Kiribati project. Thus, reducing geographic scope and focusing the available Kiribati mosquito release material into a smaller geographic area was the only practical option available to us. We have added details of the fecundity of each line under mass production conditions as a new row in Table S2

*The authors have taken the time to evaluate the insecticide resistance phenotypes which is important and relevant to the future of release programs such as this. However, they fail to discuss this in the manuscript. It would be beneficial to add a few sentences on this, particularly with respect to issues described in the Denarau area of Fiji where the authors state “active vector control activities in a largely expatriate community might have impeded wMel introgression.” Is this insecticide fogging? Larval management? The authors should also comment on the rationale behind the use of the Rockerfeller line and the exclusion of the parental Carins line from these experiments.*

Author’s response; As suggested, we have added a sentence to the Discussion on the insecticide resistance phenotypes and the vector control activities in Denarau- to the best of our knowledge this was insecticide fogging performed routinely by the private company that owns the freehold on the Denarau residential estate (Denarau Residential Estates Pty Ltd). This is done to prevent nuisance biting rather than public health reasons and is paid for by the residents through the Body Corporate.

We elected to use the Rockefeller line because it is universally accepted as a control for the insecticide phenotypic assays. We could also have used the Cairns *w*Mel parental line in place of Rockefeller. What we couldn’t do, because of the size of the experiment, was to include Cairns *w*Mel and Rockefeller, alongside the 3 x wild-type and wMel lines.

*Reviewer #3: 1. What are the economics of dengue in these hypoendemic countries?*

Author’s response; A description of the economics of dengue (and Zika and chikungunya) in these countries is outside the scope of the manuscript.

*2. What are the costs of Wolbachia release?*

Author’s response; A description of cost benefit or cost effectiveness is outside the scope of this manuscript but is the subject of work performed by Don Shepard (Brandeis University, USA) that is being prepared for publication.

*3. What is the future of the Pacific program post provision of funds from a high income government?*

Author’s response; Future *Wolbachia* deployments in the Pacific will almost certainly be dependent on donor support (philanthropy or development assistance) to low income countries (this is true for many public health interventions).

*4. Could LAMP not be transferred to local health authorities?*

Author’s response; In principal there were no technical impediments to transferring LAMP or PCR for *Wolbachia* testing to local governments; the issue was around financing the equipment, labour and reagent costs for conducting the assays on an ongoing, multiyear basis. Ultimately, there was no interest from Ministries of Health to “own” the resourcing and testing of *Wolbachia* in mosquitoes- they had other priorities in their clinical reference laboratories and public health in general and this was acutely emphasised by the Covid19 pandemic.

*5. Did COVID affect post-release monitoring? Can we really compare 2019 and before with 2020, 2021?*

Author’s response; It’s reasonable to assume that *COVID* impacted the surveillance of most notifiable diseases in the Pacific. We have been careful in not making conclusions about the public health outcomes because of the complexity of the surveillance data and the impact of *COVID* on reporting and of closed international borders, in some cases for 2 years*.* We think an evaluation 5-6 years (~2025) after establishment of *w*Mel will be informative and we intend to do this. Note we have amended the manuscript title so there’s no suggestion that we are claiming a public health outcome.

*6. Was lower fecundity in Kiribas strain real, or an artifact of lab rearing / selection?*

Author’s response; The lower fecundity of the Kiribati line was real under mass production conditions - whether it was “real” in the field (compared to local wild types) we did not try to measure and is very difficult to do in any case.

--------------------

*Reviewer #1: (No Response)*

*Reviewer #2: (No Response)*

*Reviewer #3: 1. Is Fig 8 necessary - what does it tell us?*

Author’s response; We think Figure 8 sets a useful description of chikungunya case notifications, before and after *w*Mel deployment. We don’t suggest the absence of chikungunya outbreaks since 2018 is related to *Wolbachia*.

*2. Standardise backcrossing descriptions in S2 Table.*

Author’s response; This has been done.

*3. Standardise legends, time scales etc in Fig 6 and 7*

Author’s response; We think the time scales and legends are fit for purpose for the two figures; it would not make sense to harmonise them.

--------------------

*Reviewer #1: The manuscript by Simmons et colleagues describes the introgression and establishment of populations of wMel-carrying Aedes aegypti in five countries of the Pacific island region for dengue control interventions. The program of public engagement activities and local communities involvement carried out prior and during the releases appear robust and properly assessed. The study of the basic phenotypic parameters of the newly introgressed lines is extensive, including viral challenges for assessing mosquito vector competence using four different dengue serotypes.*

*Outcomes from Wolbachia-based field interventions are important not only for expanding the application of Wolbachia in reducing dengue incidence, but also for providing significative case-based insights for releases optimization.*

*Considering the limitations and challenges imposed by the COVID-19 pandemics, the authors managed to provide a good report showing long-term monitoring data on the establishment of the released population, including a framework of epidemiological data of dengue and Chikungunya based on passive case monitoring (although most of them are not confirmed cases).*

Authors response;

*Reviewer #2: The manuscript by Simmons et al presents a timely and important evaluation of mosquito control programs, releasing Wolbachia strain wMel into wild populations to limit the spread of arboviruses such as dengue. The manuscript described in detail the necessary steps required to develop and implement a successful integration protocol in different geographical areas and is very well written. The authors do an excellent job of discussing the difficulties and breadth of community engagement required for such release programs to be successful. The authors present showing the successful introgression of wMel carrying mosquitoes into native wild population and that this appears to be sustained. They also show very preliminary data on the effect this introgression is having on dengue incidents, although as the authors point out this was severely hindered by the Sars-CoV-2 pandemic.*

*The paper is very well written and really shows the in-depth procedures required for successful release programs. The authors also do a great job of discussing the potential barriers and issues that may arise as the number of release sites increase.*

*Although a timely and important study showing the use of Wolbachia control strategies in the Pacific Island communities, there are several concerns I feel the authors must address before the paper can be accepted for publication.*

Authors response;

*Reviewer #3: A well-written, generally descriptive paper re: developments with Wolbachia release programs in the Pacific. It is of interest as an update on activities and because WHO is soon to endorse / PQ list the intervention. Title does not reflect fact that data is largely descriptive rather than statistically robust, and that releases failed in small number of areas.*

Authors response; We appreciate the positive review. We think the title is accurate; *Successful introgression of wMel Wolbachia into Aedes aegypti populations in Fiji, Vanuatu and Kiribati* accurately conveys the fact that wMel was established in the great majority of the targeted release areas.
